# Hippo-Yap Pathway Orchestrates Neural Crest Ontogenesis

**DOI:** 10.3389/fcell.2021.706623

**Published:** 2021-07-08

**Authors:** Xiaolei Zhao, Tram P. Le, Shannon Erhardt, Tina O. Findley, Jun Wang

**Affiliations:** Department of Pediatrics, McGovern Medical School, The University of Texas Health Science Center at Houston, Houston, TX, United States

**Keywords:** Hippo pathway, Yap and Taz, neural crest, migration, proliferation, differentiation

## Abstract

Neural crest (NC) cells are a migratory stem cell population in vertebrate embryogenesis that can give rise to multiple cell types, including osteoblasts, chondrocytes, smooth muscle cells, neurons, glia, and melanocytes, greatly contributing to the development of different tissues and organs. Defects in NC development are implicated in many human diseases, such as numerous syndromes, craniofacial aberration and congenital heart defects. Research on NC development has gained intense interest and made significant progress. Recent studies showed that the Hippo-Yap pathway, a conserved fundamental pathway with key roles in regulation of cell proliferation, survival, and differentiation, is indispensable for normal NC development. However, the roles and mechanisms of the Hippo-Yap pathway in NC development remain largely unknown. In this review, we summarize the key functions of the Hippo-Yap pathway indicated in NC induction, migration, proliferation, survival, and differentiation, as well as the diseases caused by its dysfunction in NC cells. We also discuss emerging current and future studies in the investigation of the Hippo-Yap pathway in NC development.

## Introduction

Neural crest (NC) is a vertebrate-specific and migratory stem cell population. It originates from the dorsal margin of the neural tube (NT) and migrates along the anterior-posterior axis of the embryo following induction (Knecht and Bronner-Fraser, 2002; Sauka-Spengler and Bronner-Fraser, 2008). In accordance with the migratory path and function, the NC mainly involves cranial NC, trunk NC, vagal NC (containing cardiac NC), and sacral NC (Noisa and Raivio, 2014). Upon reaching their terminal location, the NC cells display their remarkable multipotency, generating numerous cell types, including osteoblasts, chondrocytes, smooth muscle cells, neurons, glia, and pigment cells, and contributing to the development of different tissues/organs in a region-specific manner (Ji et al., 2019). For example, cranial NC cells display a unique ability to be induced into osteoblasts and chondrocytes, forming the majority of the bone and cartilage of the head (Johnston, 1966; Cordero et al., 2011); cardiac NC cells contribute to the formation of the aortic arch and septation of the outflow tract (aorta and pulmonary arteries) (Kirby et al., 1983; Tomita et al., 2005); trunk NC cells produce neurons and glia of the peripheral nervous system and secretory cells of the endocrine system (Serbedzija et al., 1994); sacral NC cells contribute to the development of neurons and glia of the enteric nervous system (Le Douarin et al., 2008). Defects in NC development lead to many congenital diseases, including craniofacial abnormalities, cardiovascular defects and neurological symptoms (Kirby et al., 1985; Bronner and LeDouarin, 2012). For example, Treacher Collins syndrome, a human congenital disorder caused by abnormal NC migration and differentiation, presents with mandibular hypoplasia and facial abnormalities (Sanchez et al., 2020). CHARGE syndrome is another NC formation defect-induced disorder characterized by a set of malformations including ocular coloboma, heart defects and choanal atresia (Okuno et al., 2017).

An intricate signaling network that controls NC development has been extensively studied, with great progress made in recent years. The signaling network, which includes Wnt, Sonic Hedgehog (Shh), Bmp, Fgf, Notch, and mechanical signaling, along with crosstalk among them, plays important roles in induction, specification, proliferation, migration, and differentiation of NC cells (Villanueva et al., 2002; Goldstein et al., 2005; Li et al., 2010; Manderfield et al., 2015; Chevalier et al., 2016). The Hippo-Yap pathway is also recently found indispensable for NC development (Gee et al., 2011; Hindley et al., 2016; Wang et al., 2016), but its role and related mechanisms are not well-described due to limited studies and warrant further investigation.

The Hippo signaling pathway is an evolutionarily and functionally conserved pathway involved in cell proliferation, survival, cell fate decisions and regeneration (Camargo et al., 2007; Sun et al., 2020). It contributes to the regulation of development, homeostasis, and regeneration of different tissues and organs, and its dysregulation gives rise to various diseases (Zhao et al., 2007; Lee et al., 2010; Wang et al., 2018; Zheng et al., 2020). For instance, family studies revealed that patients with heterozygous nonsense mutations in Yap1 displayed various defects including intellectual disability and orofacial clefting (Williamson et al., 2014). Anencephaly, one of the most common congenital diseases, was shown to relate with loss of UAK2 activity caused by decreased Hippo signaling via cytoplasmic Yap retention (Bonnard et al., 2020). In addition, the Cancer Genomic Atlas identified the Hippo signaling pathway as one of the 10 canonical signaling pathways frequently altered in human cancers (Sanchez-Vega et al., 2018). When Hippo signaling is on, upstream components of the Hippo signaling pathway Mst1/2 and Salv1 are phosphorylated, then activate the Lats1/2 kinases and subsequently promote the cytoplasmic degradation of the key downstream factors, Yes-associated protein (Yap) and its orthologous transcriptional coactivator with PDZ-binding motif (Taz) (Misra and Irvine, 2018). When Hippo signaling is off, Yap and Taz function as transcriptional co-activators and form a complex with transcription factors such as transcriptional enhancer activator domain (TEAD) family in the nucleus to mediate various biological activities of cells regulated by Hippo signaling (Hong and Guan, 2012; Varelas, 2014; Wang et al., 2018).

**FIGURE 1:**
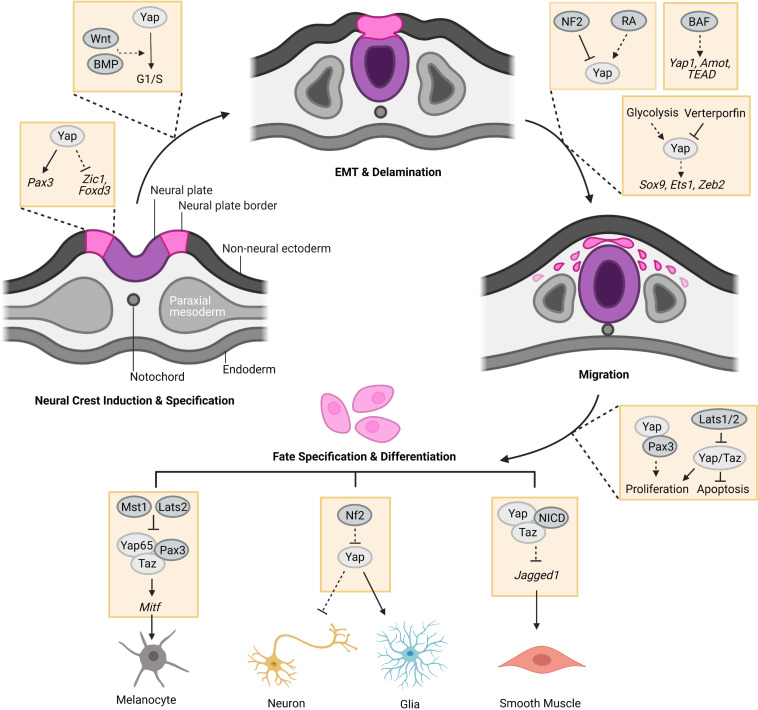
The regulation of the Hippo-Yap pathway in neural crest (NC) development. At the stage of induction, Yap holds cells in NC progenitor state by regulating the expression of NC specifier genes, such as *Pax3*, *Zic1*, and *Foxd3*. After induction and specification, Yap potentially co-regulates G1/S transition with Wnt and BMP to promote pre-migratory NC cell EMT and delamination from the neural tube for migration. Meanwhile, glycolytic flux favors migration of NC cells through enhancing YAP1-TEAD1 interaction to drive the expression of EMT factors, such as *Sox9*, *Ets1*, and *Zeb2* in chick embryos. Hippo-Yap pathway may also play a role in the regulation of BAF to NC migration in mouse embryos. In addition, Yap and Taz were found to promote proliferation and survival of NC cells which could be inhibited by Hippo kinases, Lats1/2. During NC differentiation, Yap65/Taz form a complex with Pax3 to promote melanocyte differentiation of NC cells by upregulating *Mitf* expression, which could be repressed by Hippo kinases Mst1 and Lats2. Yap overexpression favors glia cell fate of NC cells. Accordingly, Nf2 loss-of-function boosts the cell fate of glia at the expense of neurons. The complex of Yap/Taz-NICD promotes smooth muscle cell differentiation of NC cells by inhibiting the expression of *Jagged1* (Created with Biorender.com).

Recent studies indicated that the Hippo-Yap pathway is essential to NC development (Kumar et al., 2019; Bhattacharya et al., 2020), however, its role and related mechanisms are still largely unknown. In this review, we provide a research landscape on the Hippo-Yap pathway and its crosstalk with other signals in NC development shown in different experimental models (summarized in Figure 1) to provide a deeper insight into the mechanism of Hippo-Yap pathway in NC development and diseases. We also discuss the emerging areas of future studies in the Hippo-Yap regulation of NC-derived development, diseases and regeneration.

## Hippo-Yap Pathway in NC Cell Induction and Specification

Following gastrulation, NC is induced at the border between the neural plate and the non-neural ectoderm, known as the neural plate border. The induction process is orchestrated by a complex gene regulatory network that includes *Zic1*, *Gbx2*, *Pax3/7*, and *AP2* (Green et al., 2015). Among them, Pax3 and Zic1, direct upstream regulators of NC specifiers *Foxd3*, *Snail1/2*, *Twist1*, and *Tfap2b*, are both necessary and sufficient for NC cells induction (Plouhinec et al., 2014; Green et al., 2015).

Recently, there has been evidence that Yap plays a critical role in regulating *pax3* expression in the neural plate border zone in *Xenopus* embryos (Milewski et al., 2004; Gee et al., 2011). Yap gain-of-function could expand the *pax3*^+^ domain surrounding the neural plate to give rise to NC precursors in *Xenopus*, but simultaneously inhibit later NC markers expression, including *Zic1* and *FoxD3*. These observations suggested that Yap holds these cells in a NC progenitor state. *Yap* loss-of-function completely abolished *pax3* expression and resulted in loss of *pax3*^+^ NC progenitors (Gee et al., 2011). Concomitantly, a conserved Tead binding site was found in the enhancer loci of *pax3* in *Xenopus* and mice, and Tead bound to this site to activate *pax3* expression in NC (Degenhardt et al., 2010; Gee et al., 2011). Furthermore, Yap has also been shown to promote NC phenotype suggested by enriched gene expression of *SLUG, TWIST*, *AP2*, and *FOXD3*, in human pluripotent stem cells (PSC)-derived neural development studies (Hindley et al., 2016).

## Hippo-Yap Pathway in NC Cell Proliferation and Survival

Beside the important roles of induction and specification, the Hippo-Yap pathway also takes part in the significant regulation of proliferation and survival of NC cells. Previous studies established that the Hippo-Yap pathway is essential in regulating various organ growth through the control of cell proliferation and survival (Cao et al., 2008; Heallen et al., 2011; Yu et al., 2015). In instances of absent Hippo activity, Yap and Taz are localized in the nucleus, where they bind to the transcription factors, such as Tead, to promote activation of genes for proliferation (Zhao et al., 2007). While some target genes rely on the interaction of Yap and Tead for gene regulation, Manderfield and colleagues observed that within NC cells, Pax3 can act as the DNA-binding moiety for Yap, allowing for activation of pathways favoring NC proliferation (Manderfield et al., 2014).

Double conditional knock out (dCKO) of *Yap* and *Taz* in NC of mouse embryos resulted in reduced proliferation and increased apoptosis of the cells in NC-derived mandibular arch structures, at embryonic (E) 9.5 and E10.5 (Wang et al., 2016). Consistent with this observation, *Yap* and *Taz* double knockdown (dKD) O9-1 NC cells using siRNA showed reduced proliferation compared to the controls (Wang et al., 2016). In contrast, dKD of the Hippo signaling kinases Lats1/2 resulted in increased cell proliferation in O9-1 NC cells (Wang et al., 2016). In addition, Bi-Lin and colleagues observed that the loss of BAF155/170 in NC of mice embryos showed significantly decreased cell proliferation (at E10.5) and increased apoptosis (at E9.5 and 10.5) in the NT and pharyngeal arch area compared to control embryos (Bi-Lin et al., 2021). BAF complex component (Brg1) physically interacts with Hippo signaling components (Yap and Tead) in NC cells and potentially regulates many genes involved in cell proliferation and/or survival, such as *Axin2*, *Dll3*, and *Bcl10* (Bi-Lin et al., 2021). However, further studies are needed to delineate the roles of Hippo and BAF in NC proliferation and survival. Moreover, in avian embryos, the regulation of proliferation by Yap in pre-migratory NC cells was investigated by reducing *Yap* expression via electroporation of YAP shRNA construct (shYAP) in the dorsal neural tube (Kumar et al., 2019). After 24 h of transfection, shYAP NC cells showed reduced phospho-histone H3^+^ (pHH3) mitotic cells compared to control cells (Kumar et al., 2019). Altogether, Yap and Taz are essential regulators for proliferation and survival of NC cells demonstrated in both *in vivo* and *in vitro* analyses.

In contrast, the removal of *Yap* and *Taz* in NC cells (lineage labeled by tdTomato, RFP) resulted in similar proliferation rates of RFP-positive cells surrounding the third arch artery in E10.5 dKO mouse embryos compared to the controls (Manderfield et al., 2015). These inconsistent observations of Yap/Taz in proliferation rates could stem from underlying differences in NC and NC-derived cell types, a topic of interest for future studies.

## Hippo-Yap Pathway in NC Cell EMT and Migration

After induction and specification, NC cells undergo epithelial-to-mesenchymal transition (EMT) which leads to cellular architecture remodeling and adhesive property changes, along with increased cell motility. NC cells delaminate from the dorsal NT and migrate extensively throughout the embryo to contribute to the development of various tissues and organs (Basch and Bronner-Fraser, 2006; Kulesa et al., 2010). A recent study demonstrated that Yap was actively expressed in pre-migratory NC of avian embryos, and loss or gain of Yap function inhibited or promoted NC migration, respectively (Kumar et al., 2019). Reports showed that G1/S transition in the pre-migratory NC is required for NC EMT (Burstyn-Cohen and Kalcheim, 2002; Kumar et al., 2019). In avian embryos, NC cells synchronously migrated from the NT during the S phase of the cell cycle, and specific repression of the G1/S transition inhibited NC EMT and delamination both *in vivo* and in explants (Burstyn-Cohen and Kalcheim, 2002). Notably, Deepak Kumar and colleagues found that *Yap* was activated and expressed in the pre-migratory NC of avian embryos and it stimulated G1/S transition to promote pre-migratory NC determination by crosstalk with Bmp and Wnt signaling (Kumar et al., 2019). G1/S transition was inhibited in pre-migratory NC cells when Yap activity was decreased, together with reduced proliferation and increased apoptosis, which may result in the failure of pre-migratory NC cells to delaminate and migrate from the NT (Kumar et al., 2019). Concomitantly, Bmp and Wnt activations were down regulated which indicated that the proliferation and migration of the pre-migratory NC cells may be co-regulated by Yap, BMP, and Wnt, however, further study is needed (Kumar et al., 2019). Likewise, in *Yap* morphants of zebrafish embryos, cranial NC cells, identified by positive crestin expression, were shown to have significantly reduced migratory abilities resulting in abnormal distribution to the developing cranium (Jiang et al., 2009).

Hippo-Yap signaling also interacts with retinoic acid (RA) signaling to regulate NC migration. A migratory NC phenotype of multiple human neural stem cell cultures was promoted in response to the treatment of RA and upregulation of *YAP* expression by siRNA-mediated knockdown of neurofibromatosis-2 (*NF2*), an upstream regulator of Hippo kinases (Hindley et al., 2016). Notably, a recent study demonstrated that in chick embryos, NC cells underwent extensive metabolic reprogramming, increasing glucose uptake to promote NC EMT and migration (Bhattacharya et al., 2020). Glycolytic flux was found to activate Yap/Tead signaling in NC cells by enhancing YAP1-TEAD1 interaction which drove the expression of EMT factors, such as *Sox9*, *Ets1* and *Zeb2*, by interacting with tissue-specific enhancers to promote NC delamination and migration (Bhattacharya et al., 2020). Inhibition of YAP1-TEAD1 interaction with verteporfin prevented NC migration (Bhattacharya et al., 2020). In addition, a recent paper by Bi-Lin and colleagues showed that BAF deficits resulted in NC cell migration deficiencies from the pharyngeal arches to the developing outflow tract (Bi-Lin et al., 2021). Furthermore, they found that BAF complex deficiencies in NC cells resulted in downregulation of the Hippo signaling pathway including *Yap1, Tead1, Amot, and Axin2* (Bi-Lin et al., 2021); but additional studies are needed to determine the interactions between BAF and Hippo signaling for NC migration. Based on current evidence, it suggests that the Hippo-Yap pathway benefits NC cell EMT and migration.

However, by using *Wnt1-Cre*, a NC specific driver, to create a dCKO of Yap and Taz in NC cells of mice embryos, Maderfield and colleagues found that the migration of NC cells to the pharyngeal arch arteries is intact and complete in E10.5 *Yap*/*Taz* dCKO embryos, as seen in control embryos (Manderfield et al., 2015). Serial sections of fate-mapped E10.5 *Yap*/*Taz* dCKO embryos displayed regions populated by NC cells in the presumptive facial mesenchyme, peripheral nervous system and enteric ganglia, demonstrating proper migration of NC throughout the embryo (Manderfield et al., 2015). These inconsistent results may be due to varying degrees of the Hippo-Yap dependency between model systems, or delayed delamination and migration of NC cells, while indicate that the role of the Hippo-Yap pathway in NC cell migration needs to be investigated further.

## Hippo-Yap Pathway in NC Fate Specification and Differentiation

NC is a multipotent stem cell population that can generate numerous cell and tissue types after delamination from the NT. However, there are various differentiation of preferences for different types of NC cells depending on the specific regions and surrounding signals. Recent studies displayed that the Hippo-Yap pathway was involved in fate specification and differentiation of NC cells. As mentioned above, Yap interacts with Pax3 to regulate NC induction in the NT border region in *Xenopus*, while in mouse embryo, the binding of Yap65/Taz and Pax3 is indispensable for melanocyte differentiation of NC cells in a Tead-independent manner (Manderfield et al., 2014). Yap65/Taz are Pax coactivators and their deficiency in pre-migratory NC cells downregulate the expression of Pax3 target gene, *Mitf*, a critical gene required for melanogenesis. The activations of Yap65/Taz-Pax3 complex can be inhibited by Hippo kinases Mst1 and Lats2, revealing a role for Hippo signaling (Manderfield et al., 2014).

Neurons and glia in the dorsal root ganglia (DRG) are derived from NC cells. In mouse embryos, *Yap*/*Taz* are expressed in migratory NC cells, DRG progenitors and the glial lineage, but not the neuronal lineage (Serinagaoglu et al., 2015). In addition, *Yap* gain-of-function increases populations of DRG progenitors and glial cells (Serinagaoglu et al., 2015). *Nf2* loss-of-function boosts the cell fate of glia at the expense of neurons mirroring the phenotype of *Yap* gain-of-function further indicating the important roles of the Hippo-Yap signaling in NC derived neuron and glia specification.

Manderfield and colleagues found that the NC-specific deletion of *Yap*/*Taz* in mice led to vascular smooth muscle cell differentiation defects by inactivating the expression of *Jagged1*, a ligand and target of Notch signaling, by interacting with the Notch intracellular domain (NICD) (Manderfield et al., 2015). Our group also found vascular defects in *Yap*/*Taz* dCKO embryos (Wang et al., 2016). The *in vitro* O9-1 NC cells with *Yap* KO exhibited failure of smooth muscle differentiation due to Yap deficiency (Wang et al., 2016). A more recent paper showed defective smooth muscle differentiation of NC cells in pharyngeal arch arteries due to BAF155 or BAF155/170 KO in NC of E11.5 mouse embryos (Bi-Lin et al., 2021). Defective NC-derived palate mesenchyme formation was also demonstrated during secondary palate formation in BAF155 KO mouse embryos at E15.5 (Bi-Lin et al., 2021). Notably, BAF complex component (Brg1) physically interacted with Hippo signaling components (Yap and TEAD) in NC cells, potentially regulating many related genes although further studies are needed. Additionally, as a mechano-sensor of mechanical cues, Taz has been shown to promote mechanical tension-induced osteoblastic differentiation of mesenchymal stem cells (MSCs) in the rat cranial sagittal suture which are mainly derived from cranial NCCs by interacting with Rho-associated kinase (ROCK) (Li et al., 2020).

## Conclusion and Prospective

NC is characterized as a transient and multipotent stem cell population that can differentiate into various cell types to contribute to the development of diverse organs and tissues, from peripheral nervous system to craniofacial skeletal system. It is well-known that environmental cues and transcription factors orchestrate induction, EMT, migration, proliferation, survival, and differentiation of NC cells. Thus, any small perturbation may open the door for diverse diseases caused by tissues/organs defects.

The Hippo-Yap pathway is a highly conserved pathway, regulating organ size, tissue homeostasis and regeneration through the regulation of cell stemness, proliferation, survival and differentiation. Dysfunction of the Hippo-Yap pathway has been linked to an increasing number of human diseases. Recent studies revealed that the Hippo-Yap pathway also plays important roles in NC developmental processes, including the induction, proliferation, survival, EMT, migration and differentiation of NC cells (summarized in Figure 1). However, our understanding about the subject is still in the early stages due to limited studies and numerous questions regarding the role and mechanisms of the Hippo-Yap pathway in NC development and disease. For example, NC cell migration is regulated by the Hippo-Yap pathway in avian and zebrafish embryos, as well as human NC cells. However, it is not as consistently demonstrated in mouse embryos. As mentioned above, using serial sections of fate-mapped E10.5 embryos, Manderfield and colleagues found intact and complete NC migration to the pharyngeal arch arteries in *Yap*/*Taz* dCKO embryos similar to controls (Manderfield et al., 2015). Similar observations of no gross migration difference in pharyngeal arches were also found in E10.5 and E11.5 mouse embryos with BAF155/170 dCKO, which have downregulation of a part of Hippo signaling including Yap1, Tead1 and Amot in NC cells (Bi-Lin et al., 2021). Interestingly, severe migration defects were found when NC cells migrated from pharyngeal arches to the developing outflow tract of the heart in E11.5 BAF155/170 dCKO embryos. These inconsistent findings indicate that the effects of the Hippo-Yap pathway on NC migration may be influenced by species, developmental stages and types of NC cells. The possibility remains that the varying degrees of Hippo-Yap dependency observed between model systems resulted in reduced and/or delayed NC migration in the mutants. Moreover, in mouse embryos, *Yap*/*Taz* loss-offunction in NC leads to early lethality of embryos with serious neural tube regression, craniofacial malformation and vascular defects (Manderfield et al., 2015; Wang et al., 2016; Wang, 2020)]. However, the mechanisms leading to embryo lethality with various defects mentioned above require further studies.

Mechanical signaling also plays important roles in NC migration. By performing mechanical and molecular manipulations, Barriga and colleagues found that, in *Xenopus laevis* embryos, mesoderm stiffening, mainly arising from cellular density, is necessary and sufficient to trigger NC migration, and changes in substrate stiffness can cause collective cell migration by promoting EMT *in vivo* (Barriga et al., 2018). Yap/Taz are mechanosensitive mediators of mechanical cues (Dupont et al., 2011; Wei et al., 2020; Yamashiro et al., 2020). It is an interesting field for further study whether different mechanical stimulation on Yap/Taz phosphorylation and cytoplasm/nuclei translocation are responsible for the effects of mesoderm stiffness on NC EMT and migration. Hippo-Yap pathway is essential for cell proliferation and tissue regeneration (Wang et al., 2018; Zheng et al., 2020). Notably, by using a genetically dissectible mouse model of mandibular distraction osteogenesis, Ransom and colleagues revealed that mechanical stimuli could promote jaw regeneration (Ransom et al., 2018). They found that newly formed bone is clonally derived from skeleton stem cells (SSCs) which are regulated by the focal adhesion kinase (FAK) signaling pathway that transduced mechanical signaling into SSCs during distraction osteogenesis of jaw regeneration. Their data also suggested that FAK stimulates SSCs to adopt an NCC-like state by expressing NCC-associated markers such as S100a4 and Plp1, and NCC specification markers including Twist1, Tfap2a and Sox10 during distraction (Ransom et al., 2018). In addition, a recent study found that mechanical stimulus activates FAK pathway, accompanied by down-regulation of Hippo-signaling during distraction osteogenesis (Song et al., 2018). This further suggest a role of the interaction between mechanical signals and the Hippo-Yap pathways in NC development and NC-derived tissue regeneration, which is surely also an interesting field for further studies.

Taken together, these findings indicate the critical roles of the Hippo-Yap pathway in NC development, while also highlight the significance of studying the Hippo-Yap pathway in NC-related human diseases. It is also important to figure out how environmental factors coordinate with Hippo signaling to orchestrate NC development or cause NC-related diseases. In summary, the Hippo-Yap pathway has pivotal functions in NC development and warrant further investigation in the mechanisms underlying NC-related diseases with the hopes of improving current therapeutic options.

## Author Contributions

XZ, TL, SE, TF, and JW wrote the manuscript. XZ, TL, and SE made the figure. All authors contributed to the article and approved the submitted version.

## Conflict of Interest

The authors declare that the research was conducted in the absence of any commercial or financial relationships that could be construed as a potential conflict of interest.

## Abbreviations

NC: neural crest
NT: neural tube
EMT: epithelial-to-mesenchymal transition
dKD: double knock down
dCKO: double conditional knock out
RFP: Red Fluorescent Protein
RA: retinoic acid
DRG: dorsal root ganglia
NICD: Notch intracellular domain.
